# Importance and Potential of European Cross-Border Deceased Donor Organ Allocation Through FOEDUS-EOEO Platform

**DOI:** 10.3389/ti.2023.11327

**Published:** 2023-10-31

**Authors:** Andreas Elmer, Vera Valérie Lütolf, Claudia Carella, Franziska Beyeler, Nathalie Krügel, Libor Straka, Massimo Cardillo, Franz Immer

**Affiliations:** ^1^ Swisstransplant, Bern, Switzerland; ^2^ National Transplant Center, National Institute of Health (ISS), Rome, Italy; ^3^ DERS Group Ltd., Prague, Czechia

**Keywords:** organ donation, organ allocation, organ utilization, solid organ transplantation, deceased donor organ transplantation, international organ exchange

## Abstract

The FOEDUS-EOEO platform was relaunched in 2015 to allocate deceased donor organs across European borders when there are no suitable recipients in the donor’s country. We analyzed organ offers from 01.06.2015–31.12.2021 and present the number of offers and transplants, and utilization as percentage of transplanted organs. 1,483 organs were offered, 287 were transplanted (19.4% utilization). Yearly number of offers and transplants increased from 2017 to 2021, while utilization stabilized after 2018. Utilization was highest for organs offered by Slovakia (47.2%), followed for organs offered by Lithuania, France, Greece, and Czechia (19.3%–22.9%). The most frequently offered organ was the heart (n = 405; 27.3%), followed by the lungs (n = 369; 24.9%) and the liver (n = 345; 23.3%). Utilization differed significantly by organ type (highest for liver, 35.7%; followed by heart, 18.8%; and kidney, 18.3%) and by donor age (highest for 1 to 5 year-old donors (25.0%)). FOEDUS-EOEO allowed for many European patients receiving a long-awaited transplant, especially for very young pediatric patients waiting for a liver, a heart, or a kidney. The increasing number of participating countries has increased both the number of offered organs and, to a lesser extent, the number of transplanted organs.

## Graphical Abstract

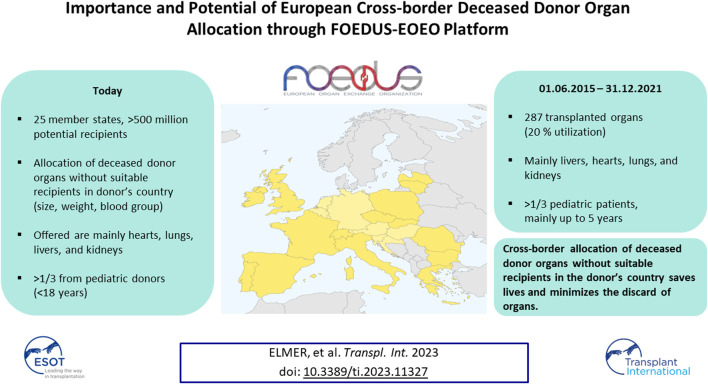

## Introduction

For organs from pediatric deceased donors (size and weight mismatch) and for organs from deceased donors with the rare blood groups AB or B, there is often no compatible recipient in the donor’s country. At the same time, pediatric or highly immunized patients often face long waiting times. Critically ill patients in urgent need of a transplant may even die on national wait lists due to the limited number of suitable organs. International collaboration allows deceased donor organ allocation to matching recipients on foreign wait lists, thus, minimizing the discard of valuable organs and increasing availability of organs for patients on national wait lists [1–4].

With the aim of optimizing HLA-matching in kidney allocation, Eurotransplant were the first to start cross-border organ allocation in Europe in the late 1960s. Since the early 1990s various bilateral agreements enabled the organ exchange between particular countries. A high mortality on the Swiss children heart wait list led Swisstransplant, the National Transplant Organization (NTO) in Switzerland, to introduce a “European Children Heart Waiting List” in 2009. Following directives 2010/53/EU [5] and 2012/25/EU [6] of the European Parliament and within the EU Action Plan on Organ Donation and Transplantation (2009–2015) [7, 8], a similar project was carried out from 2013–2016 by the European Commission and the EU member states as a so-called Joint Action [9]. An important work package of this Joint Action called “Facilitating Exchange of Organs Donated in EU Members States” (FOEDUS) was the upgrading of an existing IT platform for international allocation of national organs for which no suitable recipient can be found within the donor’s own country [10]. The platform had been formerly launched in 2012, by the EU-funded project called “Coordinating a European Initiative Among National Organizations for Organ Transplantation (COORENOR) [11, 12]. Following the end of the European Commission’s financial support for the FOEDUS Joint Action, an agreement was signed in 2016 on the initiative of 9 countries (i.e., Czechia, France, Italy, Lithuania, Poland, Romania, Slovakia, Spain and Switzerland). This agreement established a framework for cooperation, ensured the funding and maintenance of the platform and aimed to involve other European countries. The further development and continued use of the IT platform, since then called FOEDUS-EOEO platform, has accelerated communication between responsible national authorities and increased transparency and traceability of European cross-border organ allocation [13]. In consequence, the mortality on the Swiss children heart wait list decreased from over 70 percent in 2009 to below 20 percent in 2017 [14].

We analyzed all organ offers placed on the FOEDUS-EOEO platform since the operative relaunch under the responsibility of the signatory states of the cooperation agreement, on 1 June 2015, until 31 December 2021. We show how the number of offered organs and utilization developed over time and by country and we analyze how the number of offered organs and utilization vary with respect to basic donor characteristics and organ type. Based on the results, we discuss the future potential of European deceased donor organ allocation through the FOEDUS-EOEO platform after Eurotransplant joins the FOEDUS network on 1 February 2022.

## Materials and Methods

### Data

Deceased donor organ offers placed on the FOEDUS-EOEO platform from 1 June 2015 (date of the fully operational state of platform under the responsibility of the signatory states of the cooperation agreement) until 31 December 2021 were retrospectively analyzed (*n* = 1,519). A minimal electronically available dataset (including organ type, offer entry date, offer final status, donor gender, donor age, donor weight, donor height, donor blood group, donor rhesus factor, country of origin of the offer, countries which accepted the offer, and country where organ was transplanted), was made available by the Czechia based registered association responsible for managing the FOEDUS-EOEO platform. After a preliminary analysis, 36 offers were excluded because they were identified as duplicates, tests or mistaken database entries, leading to a total of *n* = 1,483 analyzed organ offers.

Further donor and organ-specific data, including some data on extended criteria, was available only on handwritten, standardized forms attached to the respective electronical records as PDFs. A digital dataset with data from these PDF forms was compiled manually by final year medical student and co-author of this article within the scope of her PhD thesis, including all offers from 1 June 2015–30 September 2020. 3.8% of those offers were from donors after cardiocirculatory death. After preliminary analysis of this additional dataset we refrained from a quantitative analysis of those variables due to data incompleteness (e.g., donation type DBD/DCD was missing in 18.9% of the cases).

### FOEDUS-EOEO Platform

The access-protected online platform is 24/7 available to all participating European NTOs and is maintained and operated by a Czech software company. It allows participating NTOs to quickly upload and simultaneously share organ offers for cross-border organ allocation, ensuring transparency and traceability in accordance with EU legislation [5, 6]. The main purpose of FOEDUS is to allocate organs for which no suitable recipient can be found within the donor’s own country. End of 2021, 17 European states were members of FOEDUS-EOEO, of which 13 actively used the platform during the study period. Since February 2022, the Eurotransplant network has also been actively using the platform after signing the cooperation agreement in 2021. Including the eight Eurotransplant member states the FOEDUS network now covers 25 states with more than 500 million inhabitants. An overview of participating states is shown in Table 1.

**TABLE 1:** FOEDUS member states, respective transplant organizations and since when they participate.

State	Transplant organization	Member since	Actively using platform since	Population	Bi-/multilateral agreement implemented partly by means of FOEDUS
Belarus	RSPC, Organ and Tissue Transplantation	2017	never used	9,340,314	-
Bulgaria	Executive Agency Medical Supervision (IAMN)	2019	2020	6,949,549	-
Czechia	Czech Transplant Coordinating Centre (KST)	2016	2015	10,693,861	Slovakia, SAT
France	Agence de la biomédecine (ABM)	2016	2015	67,197,367	Switzerland, SAT
Greece	Hellenic Transplant Organization (HTO)	2019	2017	10,696,535	Italy
Ireland	Organ Donation and Transplant Ireland	2019	never used	4,966,879	-
Italy	Italian National Transplant Centre (CNT)	2016	2015	60,286,529	Greece, SAT
Latvia	National Transplant Coordination Department - Stradini Clinical University Hospital	2019	2018	1,907,094	-
Lithuania	National Transplant Bureau under the Ministry Of Health (NTB)	2016	2015	2,793,592	-
Moldova	Transplant Agency of Moldova	2019	never used	2,573,928	-
Poland	Poltransplant	2016	2015	37,941,122	-
Portugal	Instituto Português do Sangue e da Transplantação (IPST)	2017	never used	10,291,457	SAT
Romania	National Transplant Acency (NTA)	2016	2021	19,281,118	-
Slovakia	National Transplant Organization (NTO)	2016	2015	5,457,679	Czechia
Spain	Organización Nacional de Trasplantes (ONT)	2016	2015	47,321,434	SAT
	Catalan Transplant Organization (OCATT)				
Switzerland	Swisstransplant	2016	2015	8,580,270	France, SAT
United Kingdom	NHS Blood and Transplant (NHSBT)	2017	2022	67,326,569	-
Austria, Belgium, Croatia, Germany, Hungary, Luxembourg, Netherlands, Slovenia	Eurotransplant International Foundation	2021	2022	137,501,179	-
TOTAL				511,106,476	

Note: Belarus, Ireland, Moldova, Portugal, and the United Kingdom are official member states but have never used the platform during the study period (2015–2021). If an organ offered via FOEDUS during the study period eventually had been transplanted in one of these states, the offer was counted as “not transplanted”. Population figures as provided online by The World Bank (for Belarus and Moldova; 2021 projections) and Eurostat (for all other countries; 2020 projections). SAT: South Alliance of Transplantation (Czechia, France, Italy, Portugal, Spain, Switzerland).

Only organs for which no matching recipient could be found in the donor’s country or under bilateral agreements are offered on the platform. Organ offers can either be sent simultaneously to all participating NTOs, or first to only a selection of NTOs, according to existing bi-/multilateral agreements between countries. Organs are allocated on a first-come, first-served basis. When a matching recipient is found, the bilateral organ allocation begins. It is important to note that the acceptance of a foreign organ is without any obligation on the part of the accepting country. The accepting country is explicitly under no obligation to “pay back” a received organ in return. However, the receiving country needs to organize and pay for the costs of procurement and transport.

FOEDUS-EOEO also enables urgent requests for organs when no suitable organ is available in the recipient’s country. In the present study, however, we analyzed only organ offers, not organ requests.

### Outcomes

Each offer has a final status, which can be set as “closed,” “not transplanted,” or “transplanted” by the NTO which effected the offer. Status “closed” means, an offer was not accepted by any of the NTOs which received the offer. Status “not transplanted” is meant to be chosen for organ offers initially accepted by at least one NTO, but eventually the organ was not transplanted. As there is no uniform procedure or guideline when to set the status “closed,” or “not transplanted,” we compared only offers that led to a transplanted organ (status transplanted) versus offers that did not lead to a transplanted organ (status closed and not transplanted). As the information about the receiving country in many cases is missing, we focused in our study on the organ offering and the utilization. Presented country figures always refer to the offering country, never to the receiving country.

The primary objective was to calculate utilization as the percentage of transplanted organs among all offers and to analyze whether utilization differed by offer/donor characteristics and over time. We did not investigate refusal reasons, thus utilization does only partly reflect acceptance practices of individual transplant centers.

### Statistical Analysis

As presented in Table 2, offers were divided into two groups as described above, transplanted offers (*n* = 287) vs. not transplanted offers (*n* = 1,196). Among these two groups, donor characteristics and organ types were compared for quantitative variables by using the *t*-test, or if the assumption of normality was not met, by the non-parametric Wilcoxon rank sum test. For qualitative variables Pearson’s chi-square test was used, or Fisher’s exact test in case of a small sample size. “Year” was treated as a numerical variable in the significance test.

**TABLE 2:** Organ offers placed on the FOEDUS platform from 1.6.2015 until 31.12.2021.

	Organ offers	Transplanted	Not transplanted	*p*
Total, n (%)	1,483 (100)	287 (19.4)	1,196 (80.6)	
Year of offer				
2021, n (%)	269 (18.1)	53 (19.7)	216 (80.3)	.114
2020, n (%)	256 (17.3)	50 (19.5)	206 (80.5)	
2019, n (%)	244 (16.5)	52 (21.3)	192 (78.7)	
2018, n (%)	202 (13.6)	49 (24.3)	153 (75.7)	
2017, n (%)	186 (12.5)	23 (12.4)	163 (87.6)	
2016, n (%)	230 (15.5)	40 (17.4)	190 (82.6)	
2015, n (%) only from 1.6.2015	96 (6.5)	20 (20.8)	76 (79.2)	
Organ type				
Heart, n (%)	405 (27.3)	76 (18.8)	329 (81.2)	<.001
Lungs, n (%)	369 (24.9)	43 (11.7)	326 (88.3)	
Liver, n (%)	345 (23.3)	123 (35.7)	222 (64.3)	
Kidneys, n (%)	202 (13.6)	37 (18.3)	165 (81.7)	
Small Bowel, n (%)	86 (5.8)	3 (3.5)	83 (96.5)	
Pancreas/Islets, n (%)	76 (5.1)	5 (6.6)	71 (93.4)	
Donor Characteristics				
Gender (male), n (%)	790 (53.3)	158 (55.1)	632 (52.8)	.500
Age (years), median (IQR)	34.0 (7.0–5.0)	28.0 (5.0–51.0)	35.0 (8.0–55.0)	.030
Adult group (≥18 years)	967 (65.2)	176 (18.2)	791 (81.8)	.124
Pediatric group (<18 years)	516 (34.8)	111 (21.5)	405 (78.5)	
<1 year, n (%)	128 (8.6)	21 (16.4)	107 (83.6)	.080
1–5 years, n (%)	212 (14.3)	53 (25.0)	159 (75.0)	
6–11 years, n (%)	121 (8.2)	29 (24.0)	92 (76.0)	
12–17 years, n (%)	55 (3.7)	8 (14.5)	47 (85.5)	
BMI (>20 years), median (IQR)	25.2 (22.2–28.4)	24.7 (22.0–28.0)	25.4 (22.3–28.6)	.207
Blood group				
A, n (%)	566 (38.2)	109 (19.3)	457 (80.7)	.385
0, n (%)	375 (25.3)	82 (21.9)	293 (78.1)	
AB, n (%)	274 (18.5)	45 (16.4)	229 (83.6)	
B, n (%)	268 (18.1)	51 (19.0)	217 (81.0)	

Displayed are means (±SD) and medians (IQR) for normally, and non-normally distributed numerical variables, respectively. For all categorical variables, column percentages are given in brackets in the column “Organ offers”. Except for the variable “gender”, where column percentages are given in brackets in the “Transplanted” and “Not transplanted” columns, row percentages are given in brackets, corresponding to utilization and refusal.

For the analysis, kidneys (*n* = 113) and lungs (*n* = 345) offered together were counted as one offer. The variable organ type was regrouped as follows: “kidney left” (*n* = 32), “kidney right” (*n* = 57), and “kidneys” (*n* = 113) became “Kidney”; “left lung” (*n* = 12), “right lung” (*n* = 12), and “lungs” (*n* = 345) became “Lung”; “liver” (*n* = 341), “liver left” (*n* = 2), “liver right” (*n* = 2) became “Liver”. BMI was calculated as the weight [in kilogram] divided by the square of the height [in meters], but only for donors over 20 years.

For all statistical analyses the freely available software R (version 4.2.2) was used [15].

## Results

### Organ Offers and Utilization Over Time and by Country

**FIGURE 1:**
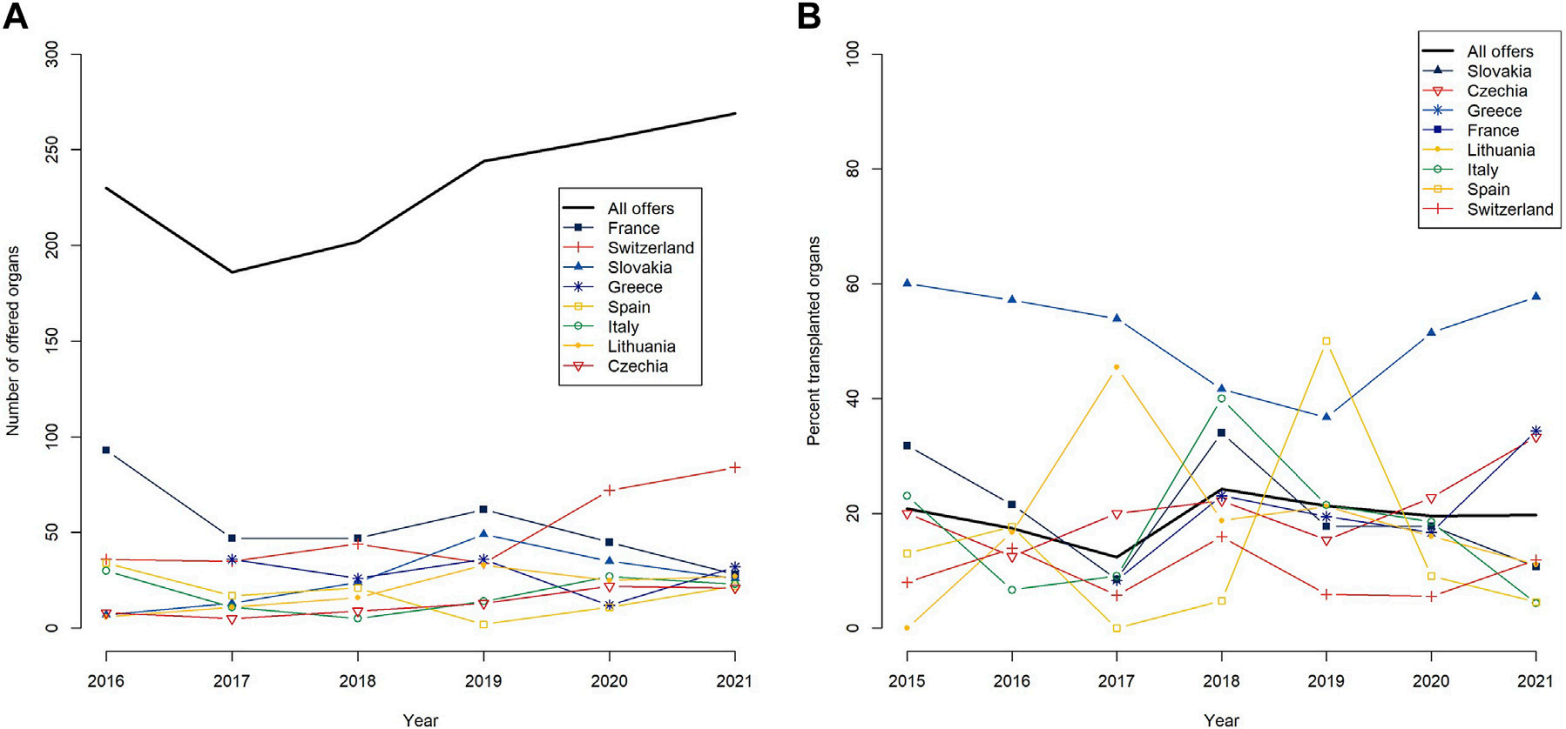
Yearly numbers of offered organs on the FOEDUS-EOEO platform from 2016–2021 **(A)**, and yearly utilization (as percent transplanted organs) since the relaunch of the platform on 1 June 2015 **(B)**, overall and for the eight countries that together effected more than 95 percent of all offers during the study period. The legends list countries in descending order of the respective metric for the entire study period.

Since the relaunch of the FOEDUS-EOEO organ allocation on 01 June 2015, 1,483 deceased donor organs were offered on the platform of which 287 were transplanted (19.4% utilization). After a sharp decrease in 2017, the yearly total number of effected offers steadily increased from 186 offers in 2017 to 269 offers in 2021 (Table 2; Figure 1A). Overall utilization per year similarly decreased until 2017, and after a maximum of 24.3% in 2018, has stabilized at just under 20% in the last 2 years of the study period (Table 2; Figure 1B).

A group of eight states together were responsible for over 95% of organ offers placed on the FOEDUS-EOEO platform during the study period, as shown in Table 3. Most organ offers were effected by France (*n* = 344 or 23.2% of total offers), followed by Switzerland (n = 330 or 22.3% of total offers). As shown in Figure 1B, state-specific organ utilization over the entire study period was highest for organs offered by Slovakia (47.2% transplanted offers). Organs offered by Lithuania, France, Greece, and Czechia had an average utilization over the entire study period (19.3%–22.9% transplanted offers). Organs offered by Switzerland, Spain, and Italy had an utilization below the average (9.7%–13.8% transplanted offers).

**TABLE 3:** Organ offers placed on the FOEDUS-EOEO platform from 1.6.2015 until 31.12.2021 according to origin state in descending order of number of offers.

State (entire study period)	Organ offers	Transplanted	Not transplanted
	n (%)	n (%)	n (%)
Total	1,483 (100)	287 (19.4)	1,196 (80.6)
France	344 (23.2)	69 (20.1)	275 (79.9)
Switzerland	330 (22.3)	32 (9.7)	298 (90.3)
Slovakia	159 (10.7	75 (47.2)	84 (52.8)
Greece	142 (9.6)	29 (20.4)	113 (79.6)
Spain	130 (8.8)	13 (10.0)	117 (90.0)
Italy	123 (8.3)	17 (13.8)	106 (86.2)
Lithuania	119 (8.0)	23 (19.3)	96 (80.7)
Czechia	83 (5.6)	19 (22.9)	64 (77.1)
*Poland*	*26 (1.8)*	*2 (7.7)*	*24 (92.3)*
*Bulgaria*	*9 (0.6)*	*3 (33.3)*	*6 (66.7)*
*Latvia*	*9 (0.6)*	*3 (33.3)*	*6 (66.7)*
*Malta*	*5 (0.3)*	*0 (0.0)*	*5 (100.0)*
*Romania*	*4 (0.3)*	*2 (50.0)*	*2 (50.0)*

The column “Organ offers” shows numbers and column percentages in brackets. The “Transplanted” and “Not transplanted” columns show numbers and row percentages in brackets, corresponding to utilization and refusal. States in italics together account for <5% of all organ offers effected in the study period and are not shown in Figure 1. Malta is not shown in Table 1 as it is not an official FOEDUS member. However, Malta has effected 5 organ offers, all from the same donor, in 2017.

### Organ Offers and Utilization by Organ Type and Donor Characteristics

The most frequently offered organ on the FOEDUS-EOEO platform was the heart (*n* = 405; 27.3%), followed by the lungs (*n* = 369; 24.9%), the liver (*n* = 345; 23.3%), the kidneys (*n* = 202; 13.6%), the small bowel (*n* = 86; 5.8%), and the pancreas/islets (*n* = 76; 5.1%). Utilization of offered livers (35.7%) was the highest and almost twice the overall average. Offered hearts (18.8%) and kidneys (18.3%) both had an average utilization, while offered lungs (11.7%), pancreas/islets (6.6%), and small intestine (3.5%) were utilized less often than the overall average. Utilization, thus, varied significantly between organ types (*p* < .001) (Table 2).

53.3 percent of effected organ offers were from male donors and the percentage of male donors was similar in transplanted (55.1%) as compared to declined offers (52.8%), respectively (*p* = .500). The median donor age of offered organs was 34 years (IQR = 7–55 years). Donors whose organs were transplanted were significantly younger than donors whose organs were declined (median age 28 vs. 35 years; *p* = .030). Thirty-five percent of organ offers (*n* = 516) were from pediatric donors under 18 years and among these pediatric offers, those from 1 to 5 year-old donors were most frequent (*n* = 212 or 14.3% of total offers). For 1 to 5 year-old donors also utilization was the highest (25%). When comparing the total pediatric donor group to the adult donor group, pediatric organs tended to be utilized more, although this was not statistically significant (21.5% vs. 18.2%; *p* = .124) (Table 2).

**FIGURE 2:**
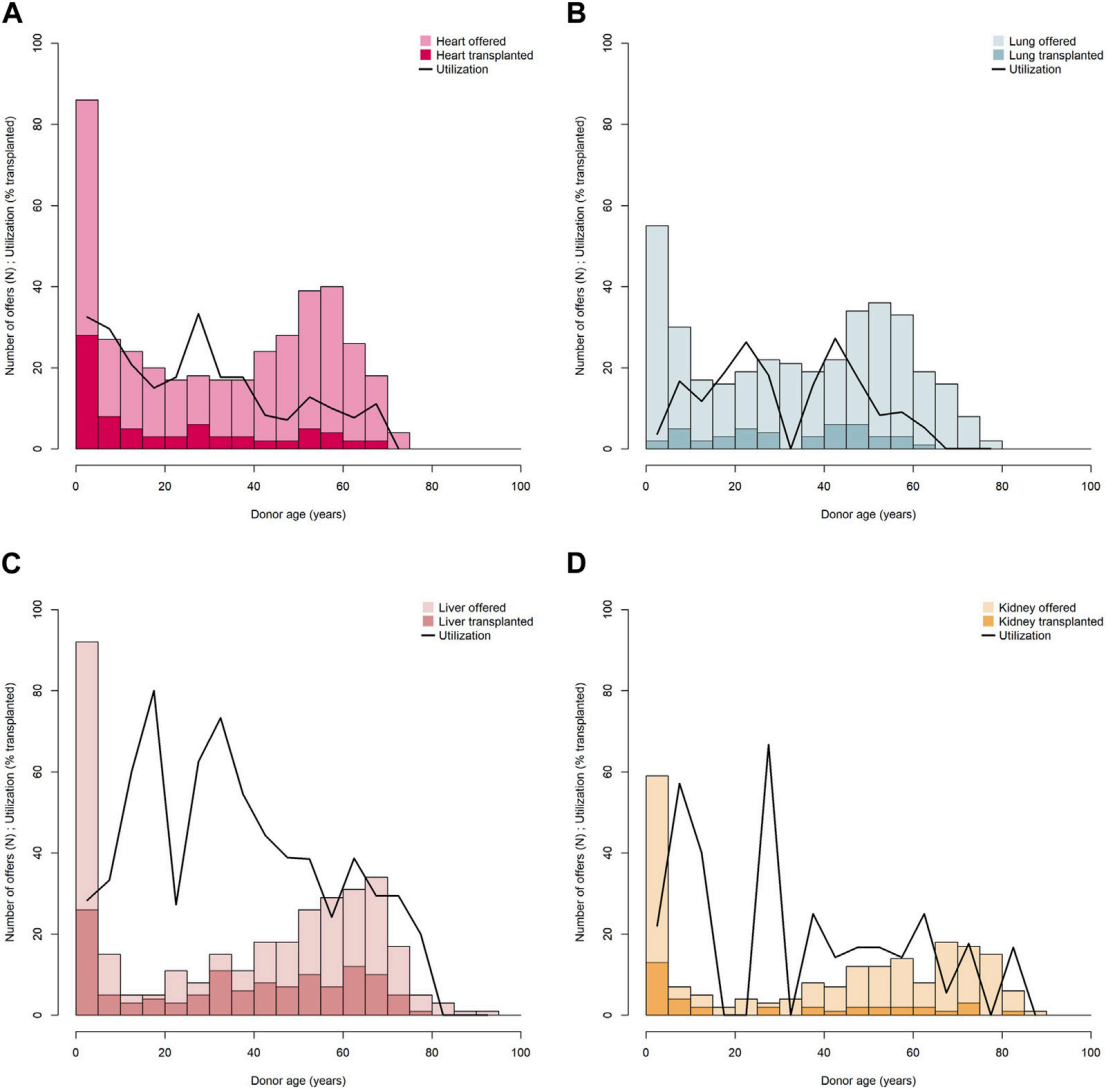
Donor age distribution and age-specific utilization of offered hearts **(A)**, lungs **(B)**, livers **(C)**, and kidneys **(D)**. Transplanted organs are shown in dark shading, utilization is shown as curved line. Age-specific utilization is calculated for each donor age category according to histogram bars (0 ≤ 5 years, >5 ≤ 10 years, >10 ≤ 15 etc.).

We looked at the age distribution of donors by organ type for all offers and for those which resulted in transplantation, and evaluated age-specific utilization (Figures 2A–D). For all organ types it applies, most offers on the FOEDUS-EOEO platform were effected in the youngest donor age group (0y–5y, first bar in histograms). In the case of the heart, the liver, and the kidney, this donor age group yielded also the most transplanted organs. In contrast, only two of 53 (4%) lung offers in the youngest donor age group were utilized. Heart and kidney utilization in the youngest donor age group was above the organ-specific average. In the case of the liver, utilization in the youngest donor age group was below average. As only three small bowels and five pancreas were transplanted in the entire study period we did not evaluate age-specific utilization for those organ types.

The median donor BMI (only donors >20 years) of all organ offers was 25.2 (IQR = 22.2–28.4) and was similar (*p* = .207) for transplanted offers (24.7; IQR = 22.0–28.0) as compared to declined offers (25.4; IQR = 22.3–28.6). Utilization was also similar across donor blood groups (*p* = .385) (Table 2).

## Discussion

### Retrospects

On the initiative of the 9 European countries Czechia, France, Italy, Lithuania, Poland, Romania, Slovakia, Spain and Switzerland, the FOEDUS-EOEO platform for European cross-border allocation of deceased donor organs was relaunched in June 2015. In 6.5 years following the relaunch, the IT platform has allowed for 287 European patients receiving a long-awaited transplant. Most of these 287 transplanted organs allocated via the FOEDUS-EOEO platform otherwise would have been discarded because of no available matching recipients on national wait lists.

There are basically two ways in an organ allocation system to allow for more patients receiving a transplant. First, by simply offering more organs. Second, by accepting more of the offered organs, thereby increasing utilization. Thanks to more countries joining FOEDUS-EOEO and actively using its IT platform, the yearly number of offered organs has increased since 2017, each year by 5–21 percent, and reached 269 offers per year in 2021. Utilization slightly decreased after 2018 and seems to have stabilized at just under 20% in 2020 and 2021. However, as long as utilization does not decline, more organs offered obviously means more transplants.

On the overall average, almost every fifth FOEDUS-EOEO organ offer is accepted and the organ utilized. When compared to other multinational organ allocation programs this is relatively low. For example, overall average utilization of organs offered by Eurotransplant is 65% [16]. To explain this discrepancy, it is worth noting that FOEDUS is intended to allocate organs when a suitable recipient cannot be found within the donor’s own country. In contrast, other European multinational organ allocation programs, such as Eurotransplant or Scandiatransplant, generally allocate organs to international recipients on a common wait list. Within FOEDUS, the exceptional high utilization of organs from Slovakia (47.2%) can partly be explained by a bilateral agreement between Czechia and Slovakia. The impact of the bilateral agreement between France and Switzerland on the allocation of livers for so-called super urgent recipients we consider negligible as with very few exceptions these allocations are processed outside the FOEDUS-EOEO platform.

The liver, however, is by far the most offered and transplanted FOEDUS organ, 123 liver transplants (or 43% of all transplants) have been facilitated through the platform, which is probably due to other bilateral agreements which are in place for this life-saving organ, in particular for pediatric liver allocation. Liver utilization is then also twice the overall average, while heart and kidney utilization are about the overall average. Lung, small bowel and pancreas offers are poorly utilized and transplanted less often. The effect of bi-/multilateral agreements (refer to Table 1) cannot be accurately determined due to differences in content (concerned organs and specific allocation rules) and varying portions of allocations managed through the FOEDUS-EOEO platform. Cross-border organ utilization, however, not only varies across organ types, but depends also on donor age, although this seems to be true only for certain organ types like the heart or the kidney. We can show that in particular very young pediatric patients (0–5 years) waiting for a liver, a heart, or a kidney transplant benefit most from cross-border organ allocation through FOEDUS-EOEO as this donor age group provides most liver, heart, and kidney offers on the platform and also most transplants.

If one compares the distribution of the blood groups among the FOEDUS-EOEO offers with average frequencies of a Caucasian population (data not shown), it is obvious that AB and B organs are overrepresented, most likely because there are fewer recipients on national wait lists for organs of these rare blood groups. It is noteworthy, however, that AB or B organs are not significantly less likely to be utilized than A or 0 organs when offered through an international platform. It appears that expanding the pool of potential recipients successfully facilitates donor-recipient matching for AB and B organs. Some may argue that organs from blood group A and 0 donors offered through an international platform are of inferior quality because national recipient pools are large and, in the case of type 0 donors, unrestricted. We were not able to thoroughly analyze organ quality in the present study, but looking at utilization, A and 0 organs were not utilized less often than average. This means that the quality of these organs was considered sufficiently good for a particular international recipient, or that the lack of a suitable national recipient in the donor’s country was not related to the overall quality of the organ.

### Prospects

**FIGURE 3:**
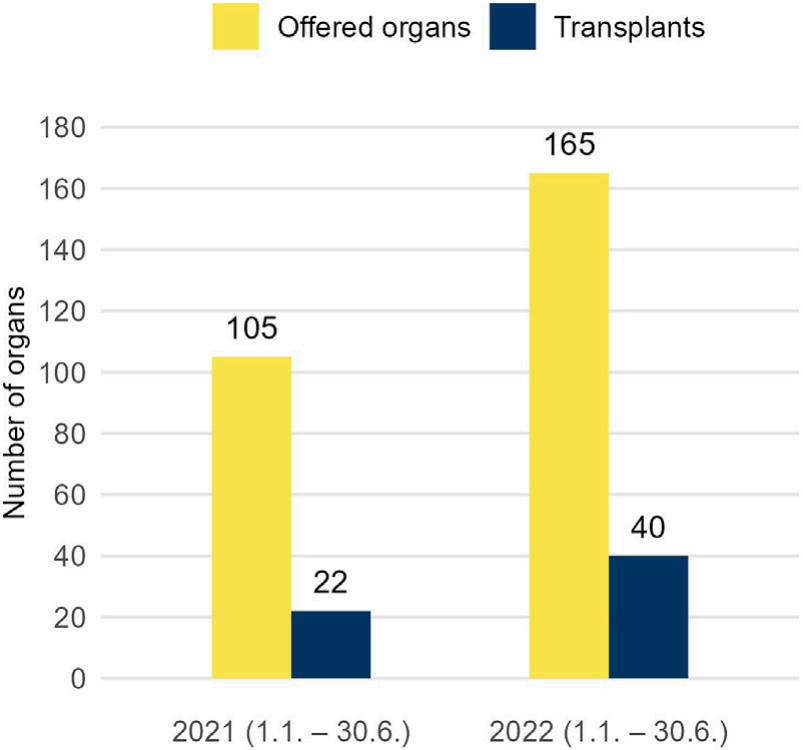
To depict the most recent development of the FOEDUS-EOEO platform after the study period, number of offered organs (left bar) and transplanted organs (right bar) in the first semester 2021 and 2022 are shown. Eurotransplant has been actively using the platform since 1 February 2022.

Most recent figures from 2022 (Figure 3) are even more encouraging. In the first semester 2022, 165 organs were offered on the FOEDUS-EOEO platform, which is a plus of 57% compared to the first semester 2021, and 40 organs were transplanted (plus 82%). Eurotransplant has been actively using the platform since February 1, 2022, making it the largest platform for cross-border organ allocation in Europe. Until end of August 2022, Eurotransplant member states in total placed alone 42 offers, which is a fifth of all offers placed in this period. Although only two Eurotransplant offers resulted in a transplant (4.8% utilization), Eurotransplant member state the Netherlands transplanted in the same period 20 organs (over one third of all transplants) offered by former FOEDUS-EOEO member states. Thus, the overall FOEDUS utilization from February to August 2022 was 25.2%, the highest since the relaunch in 2015 (Table 2).

Looking at these recent numbers it seems reasonable to forecast that participation of Eurotransplant in FOEDUS-EOEO may increase both, the number of offered organs, as well as utilization of the offered organs. Better utilization appears to be achieved primarily by Eurotransplant member states accepting more of the organs offered by former FOEDUS-EOEO member states, rather than more organs offered by Eurotransplant being transplanted in former FOEDUS-EOEO member states. If more states or EOEOs followed the Eurotransplant’s lead and joined FOEDUS-EOEO, activity could be further increased, with positive impact on pediatric patients and patients with rare blood groups who have a hard time finding a suitable donor organ.

There are, of course, other measures which could improve the platform’s utilization. For example, the fast availability of all relevant information needed for assessing organ quality is crucial. Although the use of standardized organ-specific offer forms containing key medical donor and organ information has facilitated organ evaluation for national transplant centers, our thorough analysis of these data revealed that relevant information for assessing organ quality is still missing in some offers. The forms are currently being revised and completed such that relevant information will be provided more uniformly to foreign transplant centers. Medical imaging results would be important to provide in lung offers. In the case of bilateral agreements, offering/accepting procedures must be as quick as possible to minimize loss of time when an offer subsequently is offered to all members. Other lessons learned over the years, which could serve as recommendations for regions around the world considering starting a similar collaboration, include involving users strongly during development and providing clear guidelines on how to use the system.

A major limiting factor in international organ allocation is the cold ischemia time when the organ is cold stored outside the body during transportation. Organs prone to ischemic damage are less suitable for long-distance transports. However, we believe that cooperation in legal aspects of cross-border organ allocation, together with enhanced management of organ procurement organizations, has made logistics more efficient and enabled long-distance transport also for vulnerable organs. Today, distance is practically a deciding factor only in heart allocation, but we expect, as for example, write Qin et al. in their 2022 systematic review of “Machine Perfusion for Human Heart Preservation” [17], that improvements in machine perfusion techniques may allow longer transport distances also for heart allocation in the near future. For example Swisstransplant imported two hearts in 2022 using the OCS™ Heart warm perfusion system from Rumania. As of the writing of this article Swisstransplant has imported five hearts from Czechia, France, Lithuania, and Romania in 2023.

### Strengths and Limitations of the Study

This study is the first comprehensive and long-term analysis of European cross-border organ allocation with the FOEDUS-EOEO platform–a platform which has been used for more than 10 years. To the best of our knowledge, until today only preliminary results [13] or results from a single-country perspective [11] have been published in the scientific literature. We analyzed not only activity (effected organ offers on the platform), but also utility of the platform (how many of the offered organs eventually were utilized). It could encourage European countries to participate or motivate countries in other regions of the world to set up similar programs.

Our study has also limitations. Each NTO may offer an organ either simultaneously to all participating NTOs, or, based on existing bi/multilateral agreements, first to only a selection of NTOs. This could have a significant impact on organ utilization, but we could not account for the effect size. For some NTOs it is also difficult to tell if they were actively receiving and thoroughly evaluating organ offers during the study period. Further, hearts with a maximal tolerable cold ischemia time of 4 h may not be evaluated when the donor hospital is too far away. Since the analyzed dataset does not obtain information on how many or which NTOs received and evaluated an offer, the number of potential recipients per offer remains unknown. This information, however, would help interpreting varying utilization and it would be crucial for drawing conclusions, such as if FOEDUS utilization could be increased by sending more offers to more NTOs simultaneously. Comprehensive data on donor/organ quality and refusal reasons are also important when comparing organ utilization, but in the available dataset such data were too incomplete for thorough analysis. For clarity, we treated four split livers and twelve individually offered lungs the same as whole livers and whole lungs, respectively, in our analysis. It can be argued, however, that these offers were more likely to be declined as whole liver and lung offers. Another limitation, of course, is the lack of recipient outcome data and incomplete information regarding the country of the organ’s transplantation. For the latter, FOEDUS-EOEO should improve the filing of such fundamental information in the database in the future.

## Conclusion

Over the years the FOEDUS-EOEO platform has demonstrated to be lifesaving for many European patients in need of a transplant, in particular for very young pediatric patients waiting for a liver, a heart, or a kidney transplant, or for patients waiting for a lung transplant. The increasing number of participating countries has increased both the number of offered organs and, to a lesser extent, the number of transplanted organs in Europe. In accordance with EU directives, the FOEDUS-EOEO platform ensures a high level of traceability and can be considered a best practice in European cross-border allocation of deceased donor organs for which no suitable recipient could be found under national allocation rules. To better understand and hopefully increase utilization of FOEDUS organs, more complete data on the quality of offered organs and the refusal reasons need to be analyzed. We hope that in the future the platform will be able to not only allocate those “national surplus organs”, but also allocate organs on a supranational level from the beginning for specific patient categories, such as hyperimmunized patients.

## Data Availability

The raw data supporting the conclusion of this article will be made available by the authors, without undue reservation.
